# Identification of Immune Regulatory Genes in *Apis mellifera* through Caffeine Treatment

**DOI:** 10.3390/insects11080516

**Published:** 2020-08-10

**Authors:** Yun-Heng Lu, Carol-P Wu, Cheng-Kang Tang, Yu-Hsien Lin, Houda Ouns Maaroufi, Yi-Chi Chuang, Yueh-Lung Wu

**Affiliations:** 1 Department of Entomology, National Taiwan University, Taipei 106, Taiwan; r07632017@ntu.edu.tw (Y.-H.L.); funnimax@yahoo.com (C.-P.W.); tom48836064@gmail.com (C.-K.T.); joanna60326@gmail.com (Y.-C.C.); 2 Department of Plant Physiology, Swammerdam Institute for Life Sciences, University of Amsterdam, 1098 XH Amsterdam, The Netherlands; r99632012@gmail.com; 3 Biology Centre of the Czech Academy of Science, Institute of Entomology, 37005 Ceske Budejovice, Czech Republic; houda.ouns.maaroufi@gmail.com; 4 Faculty of Science, University of South Bohemia, 37005 Ceske Budejovice, Czech Republic

**Keywords:** honeybee, deformed wing virus (DWV), caffeine, immune gene

## Abstract

**Simple Summary:**

The preference of honeybees to consume nectar with caffeine has been recorded. To investigate the effects of caffeine to this important pollinator, we first focus on the influences on immunity which is seldom explored in insects regarding caffeine. In our results, we discovered the suppressive effects on viral pathogens and the boosting effects on immunity after caffeine treatment. At least six different latent-infecting viruses in Taiwan were suppressed by caffeine. Nevertheless, the enhancement on immunity may not be effective if the bees have not been exposed to the environment or potential natural secondary metabolites like caffeine. These findings provide a basic but valuable insight into how caffeine can aid honeybees in fighting against viral invasion.

**Abstract:**

Plants and pollinators are mutually beneficial: plants provide nectar as a food source and in return their pollen is disseminated by pollinators such as honeybees. Some plants secrete chemicals to deter herbivores as a protective measure, among which is caffeine, a naturally occurring, bitter tasting, and pharmacologically active secondary compound. It can be found in low concentrations in the nectars of some plants and as such, when pollinators consume nectar, they also take in small amounts of caffeine. Whilst caffeine has been indicated as an antioxidant in both mammals and insects, the effect on insect immunity is unclear. In the present study, honeybees were treated with caffeine and the expression profiles of genes involved in immune responses were measured to evaluate the influence of caffeine on immunity. In addition, honeybees were infected with deformed wing virus (DWV) to study how caffeine affects their response against pathogens. Our results showed that caffeine can increase the expression of genes involved in immunity and reduce virus copy numbers, indicating that it has the potential to help honeybees fight against viral infection. The present study provides a valuable insight into the mechanism by which honeybees react to biotic stress and how caffeine can serve as a positive contributor, thus having a potential application in beekeeping.

## 1. Introduction

The western honeybee, *Apis mellifera*, is one of the most important pollinators in the world [1,2]. They contribute to the production of many different fruits, vegetables, crops, and aromatic seeds. However, honeybees are weak at resisting their stressors, which can cause direct/indirect injuries and affect their development, thus influencing the structure and functionality of plant-pollination networks. In recent years, the incidence rate of colony collapse disorder (CCD) [3,4], characterized by hives missing their worker bees, has increased significantly [5]; this has contributed to significant losses in the produce economy. Several factors have been suggested as contributing to this phenomenon, including poor nutrition, pesticides, pathogens, parasites, and interactions between these two factors resulting in a stressful environment for the colony [6,7,8].

Colonies suffering from CCD often exhibit signs of viral infections caused by Israel acute paralysis virus (IAPV), acute bee paralysis virus (ABPV), and deformed wing virus (DWV) [9,10,11]. DWV has been isolated in over 90% of colonies with CCD, making it the most prevalent bee virus. This RNA virus infection, spread by *Varroa destructor* mites, can cause the abnormal development of wings and memory loss since the highest titer of the virus is found in the head after infection [12,13,14]. Furthermore, DWV has a negative impact on the immune system of the honeybee [15,16]: the virus tends to negatively regulate the transcription of some genes such as dorsal 1A, a transcription factor in the family NF-κB [17,18], and the Toll pathway, which are known to target viruses [19,20,21,22]. As a result of this transcription dysregulation, transcription of antimicrobial peptides, clotting and melanization are reduced in infected honeybees [23].

Plants and pollinators benefit each other since plants provide nectar as a source of food and, in return, their pollen grains are disseminated by the visiting insect. In nectars of plants in the genera *Citrus* and *Coffea*, low concentrations of caffeine (1,3,7-trimethylxanthine) are found, which acts as an antiherbivory compound [24,25]. Previous studies have indicated that the ingestion of caffeine as nectar ingredient could increase the foraging efficiency of honeybees and thus promote pollination [26,27,28]. Furthermore, it is believed that caffeine could enhance both the learning behavior and long-term memory of forager bees [29]. In other insects, it has been found that adequate caffeine consumption influences locomotion and enhances memory; this includes studies involving hornets [*Vespa orientalis* [30]], honeybees, the green scale insect [*Coccus viridis* [31]], and flour beetles [*Tribolium castaneum* and *Tribolium confusum* [32,33]]. In addition, one study of European honeybees indicated that caffeine can enhance the tolerance to infection with the fungus parasite *Nosema ceranae* as well as extend their lifespan [34]. However, the beneficial effects of caffeine on the immune response against other pathogens, including viruses, are unknown.

Based on the above information, we hypothesized that caffeine could help the honeybee resist viral infection. We compared bees supplied with/without caffeine and assessed their immune responses against DWV infection. Furthermore, we analyzed the expression of a set of immune-related genes to determine how they are influenced by caffeine. Our results showed that the expression of immune-related genes was up-regulated after the infected honeybees were fed with caffeine, and that the replication of DWV was inhibited. These results provide evidence that caffeine is beneficial to the honeybee in fighting against pathogens in the environment. Furthermore, it provides us with a greater understanding of why bees prefer to harvest nectar from caffeine-synthesizing plants.

## 2. Materials and Methods

### 2.1. Bee Rearing

Western honeybees (*Apis mellifera*) were purchased in Hsinchu county, Taiwan, and kept at the National Taiwan University. About 50~100 bees for the experiment were collected directly from one hive as a mixed group. Then, the bees were caged in different Bugdorms (18 × 18 × 18 cm, MegaView Science, Taichung, Taiwan) at 37 °C. Prior to the experiments, bees were fed with 0.7 M sucrose water with/without caffeine (0.1 mM, Sigma-Aldrich, St. Louis, MO, USA) for one week [29,35]. One frame of the hive was taken back to the lab and incubated in 30 °C, waiting for newly emerged bees to come out. The newly emerged bees were first fed with 0.7 M sucrose water and pollen as protein source for one week to stabilize their physiological condition, then caffeine (0.1 mM) was added to the diet for another week. All food for the bees were changed every three days, and the dead bees were removed. After this feeding stage, all bees were collected for gene analysis or bioassays.

### 2.2. Deformed Wing Virus (DWV) Purification

About 80~100 bees were directly collected from their hive, and then put into cages, incubated in 30 °C. DWVs were mixed with sugar solution (0.7 M) as a regular treatment for one week. After treatment, bees were frozen in −80 °C, and then homogenized with 10 mL phosphate-buffered saline (PBS). The liquid was collected through a nylon filter to keep out residue, then we centrifuged it (16,000× *g*). Supernatant was moved to another tube and centrifuge again, then use Minisart^®^ Syringe Filters (0.45 µm) to filtrate the supernatant.

### 2.3. DWV Infection and Caffeine Treatment

Four treatment groups were prepared: control (0.7 M sucrose water only), caffeine only (0.1 mM) [29], DWV only (10^6^ virus copies), and both (caffeine/DWV). DWV was diluted with phosphate-buffered saline (PBS) to prepare a working virus solution with 5 × 10^6^ copies/µL. The sucrose water was replaced with water only one day prior to experiment and the following day the bees were forced to take 4 µL of solution mixed with 2 µL of virus and 2 µL of sucrose. After 48 h, the bees were frozen at −80 °C for total RNA extraction and gene expression analysis. After one week stabilizing the physiological condition, the bees were separated into two groups, and caffeine was then added to the diet of one of the groups for another week as the caffeine-only treatment group. Then, bees were randomly chosen from each group and forced to take 4 µL of solution mixed with 2 µL of virus and 2 µL of sucrose as virus only and both treatment group. After this feeding stage, all bees were collected for gene analysis or bioassays.

### 2.4. Total RNA Extraction

After the treatments, total RNA was extracted using a TRIzol™ reagent kit (Thermo Fisher Scientific). Whole bodies of two bees were pooled together for homogenization. RNA quantification was determined using a NanoDrop 2000 (Thermo Fisher Scientific).

### 2.5. cDNA Synthesis

cDNA synthesis of each treatment group was performed using a High-Capacity cDNA Reverse Transcription Kit (Applied Biosystems). For each sample, 2 µg of total RNA was used. The reaction was incubated in a FlexCycler 2 PCR thermal cycler (Analytic Jena AG) for 10 min at 25 °C, 120 min at 37 °C, 5 min at 85 °C, and then stopped at 4 °C. The final products were used for quantitative real-time polymerase chain reaction (RT-PCR) analysis or stored at −20 °C for later analysis.

### 2.6. Real-Time Polymerase Chain Reaction (PCR) and Data Analysis

Real-time PCR was performed using a StepOnePlus™ Real-Time PCR System (Applied Biosystems) using SensiFAST™ SYBR Hi-ROX Kit (BIOLINE, London, UK). The result (fold change) was calculated following the relative quantification theory [36]. For quantitative PCR, honeybee-specific primers for including immune, viral and carbohydrate metabolism genes (Table 1) were used as described in previous studies [35,37,38]. All samples were amplified simultaneously, and three independent experiments were performed. PCR-array images were analyzed with the software R (Version XX). Fold changes were calculated using the relative quantification method (2^−△△Ct^) [39]. Each group of tested genes was normalized to a reference gene (18s rRNA), and fold changes in the control group were used as a calibrator.

### 2.7. DWV Titer Calculation

A plasmid containing a DNA fragment of DWV was transformed to *Escherichia coli* (DH5α) and then grown in LB broth (ARROWTEC) containing ampicillin as a selection marker at 37 °C for 16 h. Plasmid extraction was conducted using a Presto™ Mini Plasmid Kit (Geneaid) and the concentration was determined using a NanoDrop 2000 (Thermo Fisher Scientific). A DNA Copy Number and Dilution Calculator (Thermo Fisher scientific website) were used to determine the amount of DNA sample equivalent to 10^10^ plasmid copies. Serial dilution was performed to prepare 10^10^ to 10^1^ plasmid copies. Real-time PCR was conducted using serially diluted plasmid samples. A standard curve was plotted using data obtained from the real-time PCR results on serially-diluted plasmid samples; regression analysis was used to calculate the virus copy numbers in the virus-treated bees.

### 2.8. Other Latent Infecting Viruses in Taiwan

Based on a previous study in Taiwan [35], we choose the also latent-infecting virus species to see if caffeine can aid the bees to resist them. The virus we use including *Vorroa destructor* virus-1 (VDV), Kashmir bee virus (KBV), Kakugo virus (KV), Israeli acute bee paralysis virus (IAPV), Sacbrood Virus (SBV), black queen cell virus (BQCV), and Chronic bee paralysis virus (CBPV) to see whether caffeine can also suppress their replication or not. After treated with caffeine, honeybees were freeze-killed and the mRNA extraction, complimentary DNA (cDNA) reverse transcription, and RT-PCR was performed to compare the amount of viral infection of honeybees treated with/without caffeine.

### 2.9. Statistical Analysis

Gene expression level results were analyzed using one-way analysis of variance (ANOVA) followed by Tukey’s post hoc test for significance using Statistica software (Version 8). The virus number was analyzed using one-way Student’s *t*-test. A *p* < 0.05 indicated a statistically significant result.

## 3. Results

### 3.1. Effects of Caffeine on Immunity Gene Signaling Factors and Anti-Microbial Peptides

**Figure 1:**
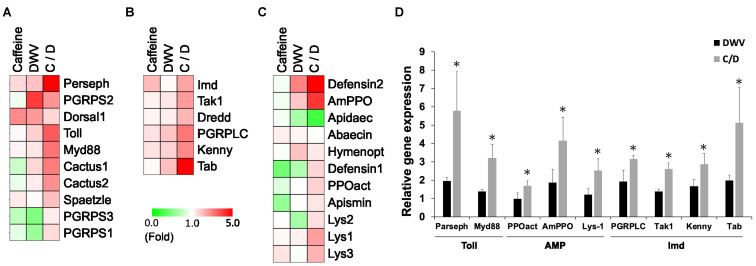
Caffeine up-regulates the expression of most genes involved in immune responses upon deformed wing virus (DWV) infection compared to control group. The relative expression rate of immune-related genes involved in (**A**) Toll pathway, (**B**) Imd pathway, and (**C**) anti-microbial peptides (AMPs). C: caffeine; DWV: deformed wing virus treatment group; C/D: caffeine treatment followed by DWV infection. The results were from real-time polymerase chain reaction (RT-PCR). Clustering analysis was based on the Euclidean distance. (**D**) Relative expression of immune-related genes with significant changes (*p* < 0.05). The values from the control groups (without any treatment or infection) were set to 1 and the values of individual genes from treatment groups were normalized accordingly. The results are from data collected from three independent experiments and presented as mean ± standard deviation (SD), *n* = 3. Statistical analysis was performed using one-way analysis of variance (ANOVA) test followed by Tukey’s post hoc test. * *p* < 0.05 as compared to the DWV infection only group.

The expression of immune response-related genes after DWV infection (Table 2), especially those involved in the Toll and Imd pathways, as well as anti-microbial peptide (AMP), were analyzed 2 days after infection. It was found that both caffeine alone (caffeine group) and DWV infection alone (DWV group) could stimulate gene expression compared with the control group, with DWV being a stronger stimulant than caffeine (Figure 1A–C). However, the highest stimulation was observed in DWV-infected bees receiving the caffeine diet prior to infection (caffeine/DWV group), which exhibited up to 5-fold stimulation in gene expression (Figure 1A–C and Table 2). Genes that exhibited more than a 1-fold increase in the DWV and caffeine/DWV groups include *parseph* and *Myd88* in the Toll signaling pathway, *PGRPLS*, *Tak1*, *Kenny*, and *Tab* in the Imd pathway, and prophenoloxidase-activating enzyme (*PPOact*), Lysozyme-1 (*Lys-1*), and AMP *defensin-2* (Figure 1D). Stimulation of these factors may provide a strong immune response against pathogen infection.

### 3.2. Caffeine Inhibits DWV Replication by Enhancing Carbohydrate Metabolism

**Figure 2:**
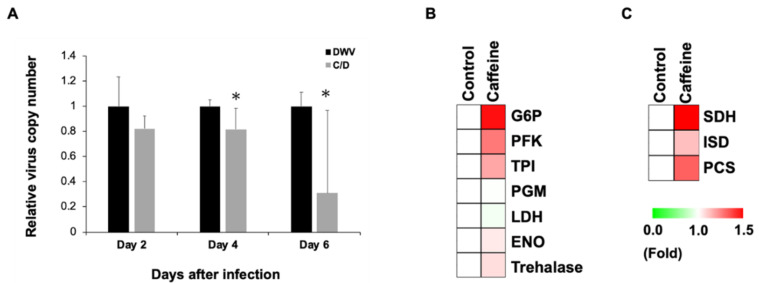
Caffeine inhibits DWV infection by promoting carbohydrate metabolism. (**A**) Relative changes in virus copy number in honeybees infected with DWV and in honeybees receiving caffeine diet prior to DWV infection. Mean and standard deviation (SD) values are shown, *n* = 6, and *p*-values were calculated using one-way Student’s *t*-test (* *p* < 0.05). WV: DWV virus infection; C/D: caffeine diet followed by DWV infection. Real-time PCR was used to quantitate the gene expression levels of metabolic enzyme genes involved in (**B**) glycolysis and (**C**) the TCA cycle after honeybees were fed with artificial feed containing caffeine. The relative expression rate of metabolic genes involved in the carbohydrate metabolism of honeybees is presented as a heat map. Clustering analysis was based on the Euclidean distance. For the caffeine treatment group in comparison to the control group, green represents a relative gene expression change <1.0 and red represents a relative gene expression change between 1.0 and 2.0.

To further investigate how caffeine stimulates the expression of immune response-related genes, the replication of DWV in infected bees with or without the caffeine diet was analyzed. It was found that the DWV genome copy number was significantly reduced in bees receiving the caffeine diet (Figure 2A), suggesting that caffeine induced the expression of immune response related genes, which in turn suppressed DWV genome replication. It is known that an optimal immune response requires a significant amount of energy [40]; therefore, we hypothesized that caffeine stimulates the expression of immune response-related genes by imposing positive effects on pathways relating to carbohydrate metabolism. To test this hypothesis, we analyzed the expression of genes involved in glycolysis and the tricarboxylic acid cycle (TCA) cycle in bees with or without the caffeine diet by real-time PCR (Table 3). Although the result had no significant difference, but still we can see the result showed the expression of genes involved in glycolysis (Figure 2B) and the TCA cycle (Figure 2C) has an up-regulated trend.

### 3.3. Caffeine also Inhibits the Infection of Other Prevalent Viruses in Taiwan

**Figure 3:**
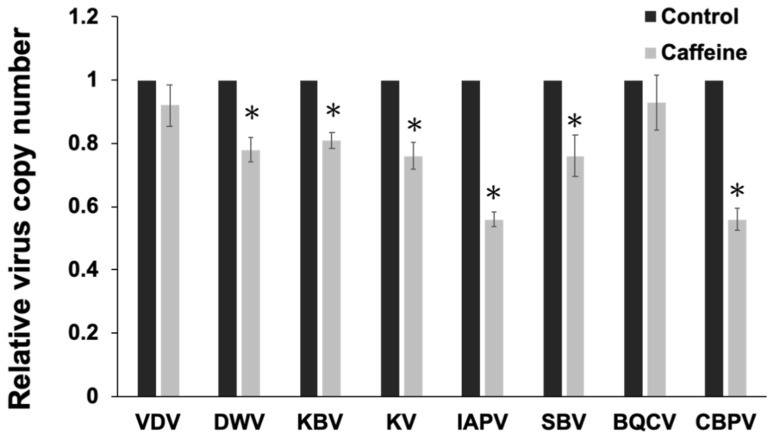
Caffeine suppresses pathogen amplification in infected honeybees of mixed group. Replicative analysis of viruses in honeybees. Relative viral DNA replication was analyzed by real-time PCR. VDV: Varroa destructor virus; DWV: deformed wing virus; KBV: Kashmir bee virus; KV: Kakugo virus; IAPV: Israel acute paralysis virus; SBV: sacbrood virus; BQCV: black queen cell virus; CBPV: chronic bee paralysis virus. The data are presented as mean ± SD, *n* = 6. Values from the untreated groups were set to 1 and the values from caffeine-treated group were adjusted accordingly. Statistical analysis was performed using the Mann–Whitney *U*-test, * *p* < 0.05 relative to data collected from the control group.

More than 8 persistent infectious viruses are prevalent among bees in Taiwan. These infected honeybees were given caffeine in their diet to evaluate its effect on viral activity in the host. Quantification of viral gene expression by real-time PCR showed a significant decrease in 6 of the viral genes in the caffeine-treated bees except for Black queen cell virus (BQCV) and Varroa destructor virus (VDV) (Figure 3). Thus, BQCV/VDV infection might not induce a noticeable immune response in honeybees.

### 3.4. Caffeine Does not Affect the Expression of Immunity Related Genes in 16-Days Old Honeybee

**Figure 4:**
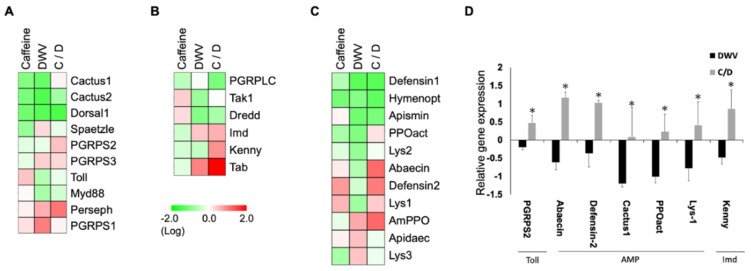
Relative expression (rER) of immune-related genes in 16-day-old adult bees. (**A**) Toll pathway, (**B**) Imd pathway, (**C**) anti-microbial peptide (AMP) (**D**) relative expression of immune-related genes with significant changes (*p* < 0.05). The scale is the logarithm of the relative fold change (control group = 0), *n* = 5. Red indicates enhanced; green indicates inhibited. C: caffeine; DWV, deformed wing virus; C/D, caffeine with DWV treatment. Clustering analysis was based on the Euclidean distance. Fold changes were compared to those in the control groups. All results were analyzed based on data collected from three independent experiments and assessed by two-way ANOVA followed by Tukey’s post hoc test. * *p* < 0.05 as compared to the DWV infection only group.

The results presented thus far were obtained from a mixture of bees from different tasks and ages. However, we also explored whether caffeine has a similar effect on 16-days old bees. For this experiment, newly-emerged bees received caffeine in their diet for 7 days and were subsequently fed with DWV. The expression of genes involved in the Toll and Imd pathways and AMPs were analyzed 2 days post-infection. It was found that the expression of genes involved in the Toll and Imd pathways were down-regulated in the DWV, caffeine, and caffeine/DWV groups (Figure 4A,B). The expression of many AMPs was also down-regulated in the DWV infection and caffeine only groups (Figure 4C). However, for several genes whose expressions were suppressed by DWV infection (including *pgrps2* and *cactus1* in the Toll signaling pathway, *kenny* in the Imd pathway, and the AMPs *abaecin and defensin-2*, and prophenoloxidase-activating enzyme (*PPOact*), and Lysozyme-1 (*Lys-1)*), the inhibitory effect could still be prevented significantly by a caffeine diet prior to infection (Figure 4D). These results indicate that for 16-day-old bees, caffeine (to which they have had no previous exposure) may have negative regulatory effects on immune response-related gene expression. This, in turn, affects the overall physiological condition of the insects in a similar manner to if the insects were exposed to an adverse environment.

## 4. Discussion

In this study, the influence of caffeine on the immune system of honeybees was evaluated. Gene expression levels were measured to demonstrate the interaction between caffeine and DWV. Although the effect of caffeine on mammals is well known [41], particularly its effects on anti-oxidation and neural activation, there are only a limited number of similar studies on insects. The results of the present study provide a basic yet valuable insight into the effect of caffeine on gene regulation in insects.

The effect of caffeine on the immune system of honeybees is almost unknown. In humans, a high concentration of caffeine can mitigate the damage caused by inflammation [42]. This is due to the inhibition on phosphodiesterase (PDE) activity, leading to an increased level of intracellular cAMP and the activation of the PKA pathway [43]. This may explain the changes in gene expression found in the present study, since the PKA pathway is also involved in regulating the immune system [44]. Analysis of the expression level of immune genes showed that they were significantly up-regulated after caffeine treatment in DWV-infected bees (Figure 1A–C), namely for *parseph* and *myd88* in the Toll pathway, *PGRPLC*, *tak1*, *kenny* and *tab* in the Imd pathway, and lysozyme-1 (*Lys-1*), prophenoloxidase-activating enzyme (*PPOact*), and the AMP gene *amPPO* (Figure 1D). Both the Toll and Imd pathways have been previously demonstrated to be involved in fighting against viral invasion in insects [45,46]. Our results also showed that caffeine treatment prior to DWV infection could sufficiently inhibit DWV infection (Figure 2). This indicates that caffeine can boost the immune system by up-regulating the expression of genes involved in pathways known to influence immune responses (such as Toll and Imd) and protect honeybees from the external stress caused by viral infections. In the results, after DWV infection, the gene expression level of the immune system is up-regulated. Although it is indicated in previous study that DWV can suppress the host’s immune system, this is the consequence of long-term and latent infection of *Vorroa destructor* and DWV [16]. In our experiment, the virus was fed to the healthy bees, so this situation is different from the previous study. This acute infection induced the spike in the immune response of honeybees against the viral invasion [47].

Until now, the effect of caffeine on gene expression in 16-day-old bees has remained unexplored. Caffeine is a natural compound present in the nectar of certain plants and, therefore, honeybees can easily consume caffeine from the environment [25,29]. Bees that are 16 days old, however, were not exposed to caffeine (and other compounds) as the foragers because they were kept in lab. The effect of a stressor and caffeine treatment was, therefore, worth investigating for these honeybees with “clean” background. Interestingly, the results are contrary to those obtained in the mixed group: caffeine does not stimulate the immune system of 16-day-old bees as it does to the mixed group in the presence of DWV infection and the expression of most genes involved in the Toll and Imd pathways and AMPs are down-regulated (Figure 4A–C).

One previous study indicated that older honeybees, such as foragers, have a high basal gene expression level related to detoxification and immune pathways for dealing with more environmental stressors [48]. This may explain different outcomes under the same experimental conditions from mixed nursing bees/forager bees and 16-day-old bees. Nevertheless, the expression of *PPOact*, *Lys-1* and *Kenny* were also significantly enhanced in DWV-infected bees, similar to in the caffeine/DWV group (Figure 4), suggesting that caffeine has a marginal boosting effect on the immune system of 16-day-old bees. The differential influences of caffeine on the immune system during pathogen infection in 16-day-old bees and older nursing bees/forager bees thus require further study.

## 5. Conclusions

In summary, caffeine has the potential to improve the resistance of honeybees to viral infection. For 16-day-old bees, caffeine has a similar effect but it is not as extensive and positive as within the mixed group. This study provides a basic knowledge of the influence of caffeine on the expression profiles of immune-related genes of honeybees. Even though the mechanism by which caffeine regulates the immune system in honeybees is not yet clear, future experiments on its effects on responses to other pathogens such as bacteria and fungi should be undertaken. This would help define a more definitive role of caffeine in promoting the immune responses of honeybees against pathogens.

## Figures and Tables

**Table 1:** List of real-time polymerase chain reaction (RT-PCR) primers and target genes.

Gene Name	Forward Sequence	Reverse Sequence
*Persephone*	CCGGTGAACTTGGAAAAGAT	ATCGCAATTTGTCCCAAAAC
Toll	TAGAGTGGCGCATTGTCAAG	ATCGCAATTTGTCCCAAAAC
Spaetzle	TGCACAAATTGTTTTTCCTGA	GTCGTCCATGAAATCGATCC
PGRPS1	TTTGAAAATTTCCTATGAAAGCA	TTTTTAATTGGTGGAGATGGAAA
PGRPS2	TAATTCATCATTCGGCGACA	TGTTTGTCCCATCCTCTTCC
PGRPS3	GAGGCTGGTACGACATTGGT	TTATAACCAGGTGCGTGTGC
PGRPLC	TCCGTCAGCCGTAGTTTTTC	CGTTTGTGCAAATCGAACAT
Myd88	TCACATCCAGATCCAACTGC	CAGCTGACGTTTGAGATTTTTG
Abaecin	CAGCATTCGCATACGTACCA	GACCAGGAAACGTTGGAAAC
Defensin-1	TGCGCTGCTAACTGTCTCAG	AATGGCACTTAACCGAAACG
Defensin-2	GCAACTACCGCCTTTACGTC	GGGTAACGTGCGACGTTTTA
Cactus-1	CACAAGATCTGGAGCAACGA	GCATTCTTGAAGGAGGAACG
Cactus-2	TTAGCAGGACAAACGGCTCT	CAGAAAGTGGTTCCGGTGTT
Dorsal-1	AAATGGTTCGCTCGTAGCAC	TCCATGATATGAGTGATGGAAA
PPOact	GTTTGGTCGACGGAAGAAAA	CCGTCGACTCGAAATCGTAT
AmPPO	AGATGGCATGCATTTGTTGA	CCACGCTCGTCTTCTTTAGG
Hymenopt	CTCTTCTGTGCCGTTGCATA	CGTCTCCTGTCATTCCATT
Apidaec	TAGTCGCGGTATTTGGGAAT	TTTCACGTGCTTCATATTCTTCA
Apisimin	TGAGCAAAATCGTTGCTGTC	AACGACATCCACGTTCGATT
Lys-1	GAACACACGGTTGGTCACTG	ATTTCCAACCATCGTTTTCG
Lys-2	CCAAATTAACAGCGCCAAGT	GCAATTCTTCACCCAACCAT
Lys-3	ATCTGTTTGCGGACCATTTC	TCGATGAATGCGAAGAAAATC
Imd	TGTTAACGACCGATGCAAAA	CATCGCTCTTTTCGGATGTT
Tak-1	ATGGATATGCTGCCAATGGT	TCGGATCGCATTCAACATAA
Dredd	GCGTCATAAAGAAAAAGGATCA	TTTCGGGTAATTGAGCAACG
Kenny	GCTGAACCAGAAAGCCACTT	TGCAAGTGATGATTGTTGGA
Tab	GCTATCATGCAGCTGTTCCA	ACACTGGGTCAGCCAATTTC
G6P	GGTTGGAGGACGTTATTC	CCATAAAGTGTGCTCCAC
PFK	ATCAGGGTATGGTAGATGGTGG	TTTTGCGACCAGCGTGAT
TPI	GAGGTTGTTGTTGGTGTACC	CCAAAAGCATAGCAGGAC
PGM	GGTACGTCATGGAGAAAGTG	TTGCGTCTCTGATTGCTT
LDH	GATGGAGGATAAATTAAAGGGAGAGA	TTGATTTTCGCGTTCCTCAAG
ENO	ACCTACAGGTGCTTCTAG	GTAGCATCAAGACCAAAC
Trehalase	ATGGAGCGGCACGAACA	GGGTCGAACGTGTCGTTGA
SDH	AGGAGGAGCGGGAAGATGTT	CAAGACCATCTCCTGTGCATGT
ISD	CATTGGCAGACATGCTCATG	TGCAATGCCAGGTCCTTT
PCS	GCAAATATTGGGAGTACGTCTATGAA	GAATCGACAGATACGGTGTTACCA

**Table 2:** Relative expression of immune-related genes in honeybees of mixed group. The results were done by real-time PCR. The values in the table represent how much percent the relative gene expression level up-/down-regulated than control group in each treatment group. Statistical analysis was done by one-way ANOVA followed by Tukey’s post hoc test. “*” indicate *p*-values < 0.05. (Caffeine/DWV compared to DWV).

**Gene Name**	**References**	**Caffeine**		**DWV**		**Caffeine/DWV**	
**Toll Pathway**		**Up**	**Dn**	**Up**	**Dn**	**Up**	**Dn**
Perseph	Hu et al., 2017	60%	-	94%	-	478% *	-
Toll	Hu et al., 2017	12%	-	73%	-	258%	-
Spaetzle	Hu et al., 2017	32%	-	8%	-	101%	-
PGRPS1	Hu et al., 2017	-	11%	-	40%	55% *	-
PGRPS2	Hu et al., 2017	-	6%	304%	-	167%	-
PGRPS3	Hu et al., 2017	-	26%	-	51%	28%	-
Myd88	Hu et al., 2017	18%	-	38%	-	220% *	-
Cactus1	Hu et al., 2017	-	21%	64%	-	203%	-
Cactus2	Hu et al., 2017	-	9%	31%	-	176%	-
Dorsal1	Hu et al., 2017	182%	-	163%	-	165%	-
**Gene Name**	**References**	**Caffeine**		**DWV**		**Caffeine/DWV**	
**Imd Pathway**		**Up**	**Dn**	**Up**	**Dn**	**Up**	**Dn**
PGRPLC	Hu et al., 2017	47%	-	92%	-	215% *	-
Imd	Hu et al., 2017	107%	-	9%	-	144%	-
Tak1	Hu et al., 2017	23%	-	39%	-	160% *	-
Dredd	Hu et al., 2017	15%	-	29%	-	115%	-
Kenny	Hu et al., 2017	43%	-	68%	-	187% *	-
Tab	Hu et al., 2017	8%	-	99%	-	413% *	-
**Gene Name**	**References**	**Caffeine**		**DWV**		**Caffeine/DWV**	
**Amp (Anti-Microbial Peptide)**		**Up**	**Dn**	**Up**	**Dn**	**Up**	**Dn**
Abaecin	Hu et al., 2017	28%	-	15%	-	-	1%
Defensin-2	Hu et al., 2017	-	4%	198%	-	2712%	-
Defensin-1	Hu et al., 2017	-	55%	-	31%	67%	-
PPOact	Hu et al., 2017	-	14%	-	-	71% *	-
Hymenopt	Hu et al., 2017	-	3%	97%	-	35%	-
AmPPO	Hu et al., 2017	-	5%	87%	-	315% *	-
Apidaec	Hu et al., 2017	-	4%	-	33%	-	75%
Apismin	Hu et al., 2017	-	42%	1%	-	40%	-
Lys-1	Hu et al., 2017	11%	-	21%	-	151% *	-
Lys-2	Hu et al., 2017	-	1%	-	35%	50%	-
Lys-3	Hu et al., 2017	42%	-	-	2%	119%	-

**Table 3:** Relative expression of carbohydrate metabolism in honeybees of mixed group. The results were done by real-time PCR. The values in the table represent how much percent the relative gene expression level up-/down-regulated than control group in caffeine only treatment group. Statistical analysis was undertaken by one-way ANOVA followed by Tukey’s *post-hoc* test. Asterisks indicate *p*-values < 0.05.

**Gene Name**	**References**	**Caffeine**	
**Glycolysis**		**Up**	**Dn**
G6P (Glucose-6-phosphate isomerase)	Froman et al., 1989	40%	-
PFK (6-phosphofructokinase)	Vora et al., 1985	26%	-
ENO (Enolase)	Marcaida et al., 2006	4%	-
PGM (Phosphoglycerate mutase)	Lu and Kreckner, 1994	-	1.4%
TPI (Triose-phosphate isomerase)	Daar et al., 1986	17%	-
Trehalase	Mori et al., 2009	6%	-
LDH (L-lactate dehydrogenase)	Chung et al., 1985	-	5%
**Gene Name**	**References**	**Caffeine**	
**TCA Cycle**		**Up**	**Dn**
SDH (Succinate dehydrogenase)	Renkema et al., 2015	51%	-
ISD (isocitrate dehydrogenase)	Ceccarelli et al., 2002	12%	-
PCS (citrate synthase)	Goldenthal et al., 1998	31%	-
